# Comparison of the review models and regulatory timelines of seven countries participating in the ECOWAS-MRH initiative: identifying opportunities for improvement

**DOI:** 10.3389/fmed.2025.1587761

**Published:** 2025-07-14

**Authors:** Mercy Owusu-Asante, Delese Mimi Darko, Seth Seaneke, Aminata Nacoulma, Oula Ibrahim Olivier Traore, Mojisola Christianah Adeyeye, Abayomi Akinyemi, Coulibaly Assane, Clarisse Épse Kaul Meledje Clamoungou, Oumy Kalsoum Ndao, Rokhaya Ndiaye Kande, James Komeh, Sheku Mansaray, Dalkoi Lamboni, Maheza Agba, Sam Salek, Stuart Walker

**Affiliations:** ^1^School of Life and Medical Sciences, University of Hertfordshire, Hatfield, United Kingdom; ^2^Food and Drugs Authority, Accra, Ghana; ^3^National Pharmaceutical Regulatory Agency, Ouagadougou, Burkina Faso; ^4^National Agency for Food and Drug Administration and Control of Nigeria, Abuja, Nigeria; ^5^Autorite Ivoirienne de Regulation Pharmaceutique (AIRP), Abidjan, Cote d’Ivoire; ^6^Senegalese Pharmaceutical Regulatory Agency (I’Agence Senegalaise de Reglementation Pharmaceutique-ARP), Dakar, Senegal; ^7^Pharmacy Board of Sierra Leone, Freetown, Sierra Leone; ^8^Medicine and Laboratories-Togo, Lome, Togo; ^9^Institute of Medicines Development, London, United Kingdom; ^10^Centre for Innovation in Regulatory Science, University of Hertfordshire, London, United Kingdom

**Keywords:** African Medicines Agency (AMA), Economic Community of West African States Medicines Regulatory Harmonisation (ECOWAS-MRH), generics, good review practices, new active substances, regulatory reliance, regulatory review models, key milestones

## Abstract

**Introduction:**

National regulatory medicines authorities (NRAs) are mandated to ensure timely access to high-quality, safe and efficacious medical products, primarily achieved through a marketing authorisation procedure established in each country. The aim of this study which was similar to that carried out in the SADC and EAC regions, was to assess and compare the review models and regulatory timelines of seven of the national medicines regulatory authorities (NRAs) of the Economic Community of West African States-Medicines Regulatory Harmonization (ECOWAS- MRH) initiative, Burkina Faso, Cote d’Ivoire, Ghana, Nigeria, Senegal, Sierra Leone and Togo, in order to identify opportunities for improvement. The NRAs were included in the study based on their active participation in the regional initiative.

**Methods:**

The Optimising Efficiencies in Regulatory Agencies (OpERA) questionnaire was completed by each of the NRAs to facilitate the assessment of the review models and regulatory timelines.

**Results:**

The authorities employ the three types of scientific review models, verification review (type 1), abridged review (type 2) and full review (type 3). Five of the NRAs deploy the fast track/priority review model in which a rapid assessment is carried out to obtain pharmacological, marketing/commercialisation, pharmacovigilance and additional clinical trial information. In Cote d’Ivoire, the priority review is used by the authority for WHO-prequalified medicines and stringent regulatory authority-approved medicines. Data requirements for the applications are essentially the same among the seven authorities. Applicants are required to provide a completed dossier in the common technical document format to support an application for marketing authorisation irrespective of the review model. Differences were noted with regard to comparison of the key features of the regulatory systems for medicines: as previously mentioned, five of the authorities required submission of a CPP with the application or before authorization. 25% of the review staff were physicians in five of the NRAs. Furthermore, procedures to allow the company response time to be measured and differentiated in the overall processing time were not available in Burkina Faso. In addition, there were differences reported in the targets for the key milestones in the full review process. These issues ultimately led to differences in the overall approval times for medicines that were processed via the full review pathway. The extent of the scientific review is dependent on the type of review model that is deployed in processing the application. Recommendations for improvement for the seven regulatory authorities include: publication of targets and timelines for key milestones; recognition of the ECOWAS-MRH initiative as a reference to expedite their approvals at the country level; and development of robust information technology systems.

**Conclusion:**

This comparative study of the review models and regulatory timelines of countries participating in the ECOWAS-MRH initiative has highlighted both the similarities among the authorities and also the differences to be addressed in order to improve upon the regulatory systems in these countries.

## 1 Introduction

National regulatory medicines authorities (NRAs) are mandated to ensure timely access to high-quality, safe and efficacious medical products. The quality assurance of medical products is primarily achieved through a scientific evaluation procedure established in each country. Ahonkai and others documented that “the mandatory individual review by multiple countries, each with its own regulatory authority, processes and capability challenges leads to increased complexity and long product approval timelines (1)” resulting in delays in making these products accessible to patients (1–6).

According to available literature “an optimized regulatory process would contribute to improved access to quality health products” (1, 7–9). Some of the contributory factors to long regulatory timelines in Sub-Saharan Africa have been identified as a “failure to leverage or rely on the findings from reviews already performed by competent authorities, disparate requirements for product approval by the countries and lengthy timelines by manufacturers to respond to regulatory queries” (1, 7–9). The discrepancy in regulatory review requirements is mainly due to additional, non-scientific factors and potentially increases regulatory timelines and presents challenges to manufacturers in their efforts to make medicines available to patients (7–10).

A reputable multinational pharmaceutical company has documented “ten pillars that represent the key hallmarks of strong regulatory review systems globally” (10). These are: strong support for regulatory convergence; guideline development and revie;, clear structure, organisation and decision making; effective application screening and review tracking mechanisms; commitment to prioritisation and transparent metrics; mechanisms for applicant-authority dialogue across the product lifecycle should be in place; transparency on marketing authorization review decisions; commitment to work-sharing, training, recognition and reliance; supportive information technology (IT) infrastructure and human resourcing; commitment to advancing regulatory science and support for innovation via regulatory data protection (10). Furthermore, it has clearly stated that “It is in the interest of all stakeholders to have effective and efficient regulatory review systems in place. From development and registration of new, innovative products for unmet medical need to the management of approved products through their life cycle, there is a pressing need to ensure streamlined regulatory review systems that result in safe and effective medicines for patients” (10).

To improve the timely access to quality medical products, some of the regulatory best practices that are being implemented by NRAs across the world include mutual recognition, reliance and other facilitated regulatory pathways (11–13). In addition, the World Health Organisation (WHO) Prequalification programme serves as a reference with regard to its implementation of facilitated regulatory pathways to benefit low- and middle-income countries (3).

In 2020, the United States Food and Drug Administration (FDA) updated its generic drug application prioritisation policy “to efficiently allocate limited agency resources to areas where priority review is most likely to meaningfully increase generic drug access and ensure fairness to applicants” (14). In Europe the European Medicines Agency (EMA) has a procedure in place to accelerate assessment of marketing authorisation applications that can impact public health (15).

The World Health Organisation reported that the absence of well-functioning regulatory systems to facilitate timely access to quality, safe and efficacious medical products was clearly evident during the COVID-19 pandemic (16).

Insufficient publicly available information on the differences and similarities among NRAs has also been reported, even at the global level, making it very challenging to achieve efficient national regulatory systems. Thoroughly investigating the critical components of regulatory systems will help to discover their current state and propose appropriate improvements to address any identified gaps (1, 2, 10).

Ahonkhai and colleagues proposed that future publications should pay attention to the outcome of implementation of various regulatory measures for NRAs in Sub-Saharan Africa to achieve shorter timelines (1). Furthermore, Alfonso and associates accurately stated that “regulatory system strengthening via regional coordination could also support the operationalization of a newly formed continental authority, the African Medicines Agency (AMA)” (10). Therefore, this study of the review models and regulatory timelines used in the Economic Community of West African States Medicines Regulatory Harmonisation (ECOWAS- MRH) initiative is considered timely.

According to the WHO “good communication is critical and has many advantages for regulatory authorities, applicants and the public. It can improve the efficiency of the development and review processes and thus ultimately speed up patient access to quality medical products” (17). As part of that good communication, NRAs have been urged to share their best practices to enhance efficiency in the review process of medicines (10).

In light of the successful assessment of the review models and regulatory timelines of countries participating in the ZaZiBoNa and EAC-MRH initiatives (18, 19), it is apt that the review models and regulatory timelines of countries participating in the ECOWAS-MRH initiative are assessed and hence the implementation of this study. The study is therefore aimed at assessing the review models and regulatory timelines of countries participating in the ECOWAS-MRH initiative and to communicate the findings to other regulatory authorities, stakeholders and the public and serve as a reference for future comparative analyses across the NRAs in ECOWAS to establish best practices.

This publication, the second of a two-part series, provides an insight into the review models and regulatory timelines of countries participating in the ECOWAS-MRH initiative, while the first publication compared the countries’ good review practices (20).

## 2 Materials and methods

### 2.1 Study participants

All seven active NRAs of the ECOWAS-MRH initiative, namely National Pharmaceutical Regulatory Agency-Burkina Faso; Autorite Ivoirienne de Regulation Pharmaceutique (AIRP) -Republic of Cote d’Ivoire; Food and Drugs Authority (Ghana-FDA); National Agency for Food and Drug Administration and Control (NAFDAC)- The Federal Republic of Nigeria; Senegalese Pharmaceutical Regulatory Agency (I’Agence Senegalaise de Reglementation Pharmaceutique -ARP) - Republic of Senegal; Pharmacy Board of Sierra Leone (PBSL) and the Directorate of Pharmacy, Medicine and Laboratories-Togo, participated in the study between August 2021 and December 2024.

### 2.2 Data collection

The Optimising Efficiencies in Regulatory Agencies (OpERA) questionnaire, which had been developed by the Centre for Innovation in Regulatory Science (CIRS) (21) was completed by each of the NRAs under the supervision of the Head of the Agency. The OpERA questionnaire was divided into three sections to address metrics for new active substances (NASs), generics, WHO prequalified generics in part 1, types of review models and extent of scientific assessment in part 2 and key milestones in part 3.

## 3 Results

For the purpose of clarity, the results of the study will be presented in three parts: Part (1) metrics for new active substance (NASs), generics and WHO-prequalified generics received and approved in 2023; Part (2) types of review models and extent of scientific assessment and Part (3) key milestones in the review process.

### 3.1 Part 1. Metrics of NASs, generics and WHO-prequalified generics

A comparison of metrics for NASs, generics and WHO-prequalified generics that were received and approved in 2023 is provided in Table 1. It is noted that a large number of generics were not approved by the authorities. This is very concerning and requires attention of both manufacturers and the regulators.

**Table 1:** Comparison of metrics for NASs, generics and WHO-prequalified generics in 2023.

Country	Burkina Faso	Cote d’Ivoire	Ghana	Nigeria	Senegal	Sierra Leone	Togo
NASs							
Received	NA	23	26	NA	NA	4	NA
Approved	NA	23	17	1	NA	4	NA
Generics							
Received	NA	312	1,189	NA	NA	550	NA
Approved	NA	90	577	729	NA	390	NA
WHO-prequalified generics							
Received	NA	21	3	NA	NA	2	NA
Approved	NA	21	3	8	NA	2	NA

NASs, new active substances; NA, not available.

### 3.2 Mean approval times

Four out of the seven NRAs provided data regarding their mean approval times (calendar days) for NASs, generics and WHO-prequalified generics in 2023. Cote d’Ivoire, reported the highest mean approval time of 240 calendar days for NASs that were processed via the full review pathway. Nigeria on the other hand reported the longest mean approval time of 247 calendar days with regard to generics that were processed via the full review pathway. For applications that were processed via the abridged review pathway, Ghana reported the highest mean approval time of 116 calendar days with regard to both NASs and generics. Finally for applications that were processed via the verification pathway, Ghana reported the highest mean approval time (Table 2).

**Table 2:** Comparison of mean approval times (calendar days) for NASs, generics and WHO- prequalified generics in 2023.

Country	Burkina Faso	Cote d’Ivoire	Ghana	Nigeria	Senegal	Sierra Leone	Togo
Full review							
NASs	NA	240	56	NA	NA	150	NA
Generics	NA	240	56	247	NA	150	NA
WHO PQ generics	NA	NA	NA*	NA*	NA	75	NA
Abridged							
NASs	NA	NA	116	30	NA	75	NA
Generics	NA	NA	116	NA	NA	53	NA
WHO PQ generics	NA	NA	NA*	NA	NA	30	NA
Verification							
NASs	NA	NA	NA	NA	NA	53	NA
Generics	NA	NA	NA*	NA*	NA	38	NA
WHO PQ generics	NA	NA	118	60	NA	30	NA

NASs, new active substances; PQ, prequalified. NA, Not available; NA*, Not applicable.

It is noted that the mean approval timelines were met for NAS and generics that were processed via the full review pathway by Ghana in 2023, however it exceeded the approval timeline for WHO-prequalified generics, NAS and other generics that were processed via the verification and abridged review pathways, respectively, by about 30 days.

### 3.3 Part 2. Types of review models and extent of scientific assessment

The authorities employ three types of scientific review models, verification review (type 1), abridged review (type 2) and full review (type 3). The verification model is used for applications that have been authorised by one or more recognised reference or benchmark authorities. The definition of a recognised reference authority is dependent on each NRA. Notwithstanding, generally the recognised reference authorities are the WHO, EMA, United States FDA, Health Canada, TGA Australia and Swissmedic. The NRA in the importing country verifies that the product’s quality, safety and efficacy in both the reference and importing countries are essentially the same. By employing this model, applications are reviewed within a short time, usually within 90 days.

The NRAs involved in this study participate in the joint regulatory review process and recognise recommendations made at the ECOWAS regional level.

The abridged model is used for applications that have been authorised by a recognised reference authority, and requires an abridged independent review of the quality data, which may be relevant to stability zone IVb conditions. A benefit–risk assessment may also be undertaken with regard to its use in the importing country.

The full review model is used for applications that have not been authorised by a recognised reference authority and therefore requires a full review of the product’s quality, safety and efficacy. In addition to the three types of review models defined above, the authorities have a fast track/ priority review model in place for prioritising applications for unmet medical needs / public health programmes in each country (Table 3).

**Table 3:** Review models employed and target timelines (calendar days).

Type of review model	Burkina Faso	Cote d’Ivoire	Ghana (excludes applicant time)	Nigeria	Senegal	Sierra Leone	Togo
Verification review (Type 1)	✓		✓	✓	✓	✓	
Target	NA	NA*	90	90	90	NA	NA*
Abridged review (Type 2)	✓	✓	✓	✓	✓	✓	✓
Target	NA	NA	90	NA	NA	NA	NA
Full review (Type 3)	✓	✓	✓	✓	✓	✓	✓
Target	NA	NA	180	240	120	NA	NA
Fast track/priority review	✓	✓	✓	✓		✓	
Target	NA	NA	90	NA	NA*	NA	NA*

NA, not available; NA*, not applicable.

#### 3.3.1 Verification review

Five of the NRAs deploy the verification review model. Applications submitted through the WHO collaborative procedures and the Market Authorisation for Global Health Products (MAGHP) by Swissmedic are processed under the verification review model. The verification process is used to validate the status of the product and ensure that the product intended for local marketing conforms to the authorised product. In Nigeria and Sierra Leone, applications that have been assessed by stringent regulatory authorities (SRAs), members of the International Council for Harmonisation of Technical Requirements for Pharmaceuticals for Human Use (ICH) and the West African Health Organisation (WAHO) are also processed through the verification review model. Authorities that have achieved WHO Global Benchmarking Tool (GBT) maturity level 3 or 4 are recognised as reference authorities (13). In some instance a checklist is used to confirm the completeness of the data. Unredacted assessment reports are required from these reference authorities.

#### 3.3.2 Abridged review

All seven of the NRAs deploy the abridged review model. In Ghana, applications previously registered by an SRA (EMA, USFDA, the United Kingdom Medicines and Healthcare products Regulatory Agency [MHRA] and Health Canada) are assessed via the abridged review pathway. An abridged assessment is carried out in relation to the use of the products under local/ national conditions. In Togo, products approved by SRAs and WHO-prequalified medicines are assessed via the abridged review pathway (Table 3).

#### 3.3.3 Full review

All the NRAs deploy the full review model. The authorities are capable of carrying out full assessment of quality, pre-clinical (safety) and clinical (efficacy) data. Information on prior registration elsewhere may still be a pre-requisite to final authorisation if not a full review will be carried out. Generally, applications for medicines from non-ICH regions and non-WHO prequalified products are processed via this pathway (Table 3).

#### 3.3.4 Fast track/priority review

The NRAs of Burkina Faso, Cote d’Ivoire, Ghana, Nigeria and Sierra Leone deploy the fast track/priority review model.

Under this pathway a rapid assessment is carried out to obtain pharmacological, marketing/commercialisation, pharmacovigilance and additional clinical trials information. In Cote d’Ivoire, priority review is used by the authority for WHO-prequalified medicines and SRA-approved medicines (Table 3).

#### 3.3.5 Data requirements and key features of assessment

A summary comparison of key features of the regulatory systems for processing applications for marketing authorisation for medicines in the NRAs is provided in Table 4. It is noted that there are several similarities in the regulatory systems of these authorities.

**Table 4:** Key features of the regulatory systems for medicines.

Marketing authorisations	Burkina Faso	Cote d’Ivoire	Ghana	Nigeria	Senegal	Sierra Leone	Togo
Certificate of a Pharmaceutical Product (CPP): CPP required with application or before authorisation is issued	✓		✓	✓	✓	✓	
Common Technical Document (CTD): CTD format mandatory for applications	✓	✓	✓	✓	✓	✓	✓
Medical staff: More than 25% within the authority review staff are physicians	✓			✓	✓	✓	✓
Review times: Authority sets targets for time spent on scientific assessment of NASs and generic applications	✓	✓	✓	✓	✓	✓	✓
Approval times: Authority has target for the overall time for the review and approval of an application	✓	✓	✓	✓	✓	✓	✓
Questions to sponsors are batched at fixed points in the review procedure	✓	✓	✓	✓	✓	✓	✓
Company response time: Recording procedures allow the company response time to be measured and differentiated in the overall processing time		✓	✓	✓	✓	✓	✓
Priority reviews: The agency recognises medical urgency as a criterion for accelerating the review and approval process for qualifying products	✓	✓	✓	✓	✓	✓	✓
Parallel processing: Different sections of technical data reviewed in parallel rather than sequentially	✓	✓	✓	✓	✓	✓	✓
Price negotiation: Discussion of pricing separate from the technical review and does not delay the approval of products	✓	✓	✓	✓	✓	✓	✓
Sample analysis: The focus is on checking quality in the marketplace and requirements for analytical work do not delay the marketing authorisation	✓	✓	✓	✓	✓	✓	✓

Differences included the fact that five of the authorities except Cote d’Ivoire and Togo required submission of a WHO certificate of a pharmaceutical product (CPP) with the application or before authorisation is issued. 25% of the authority review staff were physicians in five of the authorities with the exception of Ghana and Nigeria. It is suggested that pharmacists rather than physicians are better equipped with the requisite knowledge to assess the quality part of medicinal product dossiers. However, physicians can better handle the safety and efficacy parts of the dossiers. Therefore, best practice would be to have a good balance of pharmacists and physicians to ensure efficiency and effectiveness in the review of medicinal product dossiers.

Recording procedures to allow the company response time to be measured and differentiated in the overall processing time were not available in Burkina Faso.

The data requirements for the applications are essentially the same among the authorities. Applicants are required to provide a completed dossier in the ICH common technical document (CTD) format to support an application for marketing authorisation/registration, irrespective of the review model to be deployed in processing the application. Each authority sets targets for the time it spends on the scientific assessment of NAS and generic applications. Additionally, each authority has a target for the overall time for the review and approval of an application. Questions to sponsors are batched at fixed points in the review procedure. Each authority recognises medical urgency as a criterion for accelerating the review and approval process for qualifying products. Different sections of the technical data are reviewed in parallel rather than sequentially. Discussion of pricing is separate from the technical review and does not delay the approval of products. The focus of each authority is on checking quality in the marketplace and requirements for analytical work do not delay the marketing authorisation.

The key parameters of the scientific review is dependent on the type of review model that is deployed in processing the application. Since all the NRAs deploy the full review model, Table 5 shows a comparison of the key parameters of full scientific assessment.

**Table 5:** Key parameters of full scientific assessment.

Parameter	Burkina Faso	Cote d’Ivoire	Ghana	Nigeria	Senegal	Sierra Leone	Togo
Chemistry, manufacturing and control (CMC) data extensive assessment	✓	✓	✓	✓	✓	✓	✓
Non-clinical data extensive assessment	✓	✓	✓	✓^a^	✓	✓	✓
Clinical data extensive assessment	✓	✓	✓	✓	✓	✓	✓
Bioequivalence data extensive assessment	✓	✓	✓	✓	✓	✓	✓
Additional information obtained (where appropriate)	✓	✓	✓	✓	✓	✓	✓
Other agencies internal review reports	✓	✓	✓	✓	✓	✓	✓
Medical and scientific literature	✓	✓	✓	✓	✓	✓	✓

✓^a^ Required for NAS but not generic products.

### 3.4 Part 3. Key milestones in the review process

**Figure 1:**
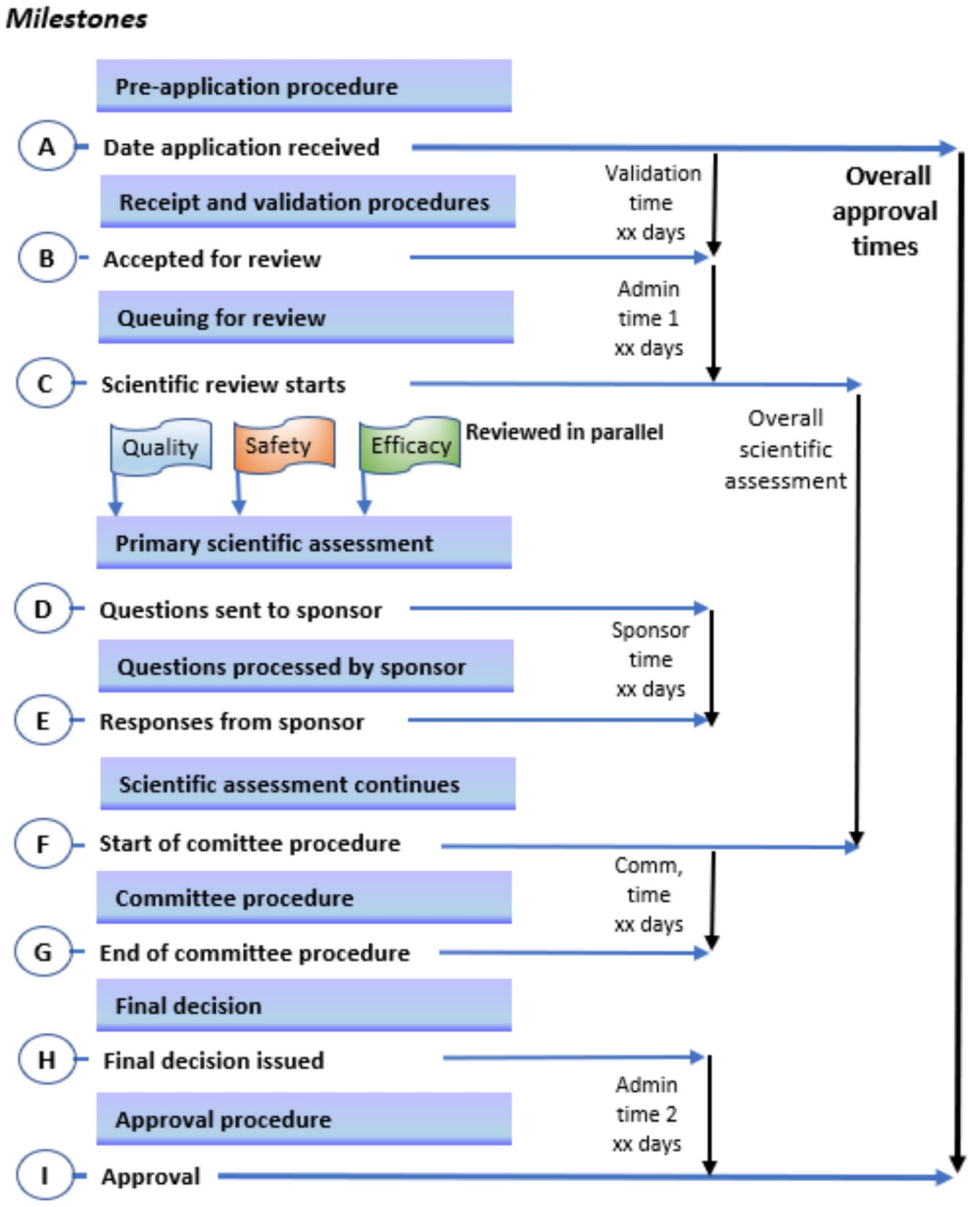
Key milestones in the review process. A status map of the review process and authorisation of a product approved on the first cycle (that is, does not include a second or more cycles for products approved subject to the submission of additional data) for a typical national medical regulatory authority with maturity level 3; this figure uses a format that correlates with key milestones of the review process.

A status map of the review process and authorisation of a product approved on the first cycle (that is, does not include a second or more cycles for products approved subject to the submission of additional data) for a typical NRA with maturity level 3 is provided in Figure 1. This figure uses a format that correlates with key milestones of the review process. It is noted that the NRAs have identified similar key milestones in their full review pathways; that is, receipt and validation; queuing; primary scientific assessment; questions to applicant; review by expert committee and approval procedure. Table 6 shows a comparison of targets for key milestones in the full (type 3) review process.

**Table 6:** Comparison of targets for key milestones in the full (type 3) review process (calendar days).

Target	Burkina Faso	Cote d’Ivoire	Ghana	Nigeria	Senegal	Sierra Leone	Togo
Receipt and validation (A-B)	15	30	28	10	1	7	NA
Queuing (B-C)	14–56	14–56	14–42	NA	NA	14–56	NA
Primary scientific assessment (C-D)	15	30	112	80	NA	NA	NA
Questions to applicant (clock stop) (D-E)	180	90	30	90	NA	42	90
Review by Expert Committee (G-H)	15	20–30	1	30	NA	7	1–2
Approval procedure (Admin)	<30	30–90	30	30	30–90	30	NA
Overall approval time (A-I)	311 (excludes applicant’s time)	120 (excludes applicant’s time)	180 (excludes applicant’s time)	240 (excludes applicant’s time)	120 (excludes applicant’s time)	180 (excludes applicant’s time)	120 (excludes applicant’s time)

NA, Not available.

#### 3.4.1 Receipt and validation

There is no formal procedure before the start of the application process. The receipt and **v**alidation process lasts from 1 to 30 days. The variation in the time is dependent on the initial administrative and technical processes that are in place in the NRAs. Applications are screened to ascertain their level of completeness in order to be processed for assessment. For incomplete applications, a request for the missing data is sent to the applicant, who is obliged to provide a satisfactory response within a stipulated time limit.

#### 3.4.2 Queue time

Applications that are not eligible for the fast track/priority pathway are placed in a queue according to their review pathways to await their turn for the primary scientific assessment. The queue time varies from 14 to 56 days among the authorities. The queue time is dependent on the authority workload and availability of assessors to conduct the primary scientific assessment. In Ghana, samples of applications are sent to the FDA Ghana laboratory for analysis while the dossier is placed in the queue for assessment. Some of the authorities regard backlog as a challenge and try to address this by increasing the uptake of assessors, introducing smart approaches such as not duplicating effort on same dossier submitted by a different applicant from the same manufacturer and implementing risk-based assessment. Additionally, to improve the efficiency of the review process, Nigeria applies product review performance metrics versus the volume of applications received.

#### 3.4.3 Primary scientific assessment

The duration of the primary scientific assessment varies from 15 to 112 days among the authorities. (Table 4) with the different sections of the technical data being reviewed in parallel rather than sequentially. The time spent on the assessment of dossiers is very much dependent on the technical expertise, knowledge and experience of assessors. The assessment is carried out by the authority’s technical staff; however, some of the NRAs use external experts to assess clinical and non-clinical data.

#### 3.4.4 Questions to applicants

Following completion of the primary scientific assessment, questions are sent to applicants for response to be provided within a time frame of between 30 to 180 days. Depending on the NMRA, questions are collected into a single batch and sent either prior to the expert committee meeting or after the expert committee has reviewed them. The NRAs except Sierra Leone have a provision for applicants to hold meetings with the authority staff to discuss questions and queries that arise during the assessment. In the NRAs except Sierra Leone, the scientific review ceases (a “clock stop”) while questions are being processed by the applicant.

#### 3.4.5 Review by expert committee

A committee of experts is consulted in the review process after the authorities have reviewed and reported on the scientific data. In Burkina Faso, a Scientific Committee include 3 external experts who are incorporated into the internal and external scientific evaluation process of the agency depending on the product that is being evaluated (medicines, vaccines, biological products, alimentary supplements and medical devices). The Expert Committee takes between 1 and 30 days to review the application, dossier assessment and laboratory analytical report, and makes a final decision on applications for marketing authorisation. There is no additional step in the scientific review process after the Committee has given its opinion.

#### 3.4.6 Authorisation procedure

The NRAs take between 30 and 90 days to grant approval after receiving a positive outcome from the expert Committee.

## 4 Discussion

This study compared the review models and regulatory timelines of countries that actively participate in the ECOWAS-MRH initiative in order to identify opportunities for improvement.

This study, which is similar to the SADC and EAC regional studies by Sithole and colleagues (18) and Ngum and associates (19), also sought additionally to identify the similarities and differences among these NRAs as they work together to advance the course of the ECOWAS-MRH initiative.

The authorities generally utilise the standard review pathways and have also set realistic target timelines with regard to the limited resources available in West Africa. This is a significant observation for the NRAs, in that it indicates that these authorities are operating in similar ways as recognised reference authorities (19). By implementing verification and abridged review pathways, it can be inferred that these authorities “leverage or rely on findings from reviews already performed by competent authorities” (1).

The issue of “disparate requirements for product approval by the countries,” which was previously reported by Ahonkai et al. (1) have now been addressed by the NRAs embracing/implementing reliance models as emphasised by the WHO (13) and AMRH/AMA., There were more similarities observed among the authorities regarding data requirements and extent of assessment of the scientific data as this is largely due to the fact the submission of documentation is in the CTD format.

Differences were noted with regard to comparison of the key features of the regulatory systems for medicines: as previously mentioned, five of the authorities required submission of a CPP with the application or before authorisation, more than 25% of the review staff were physicians in five of the NRAs. Furthermore, procedures to allow the company response time to be measured and differentiated in the overall processing time were not available in Burkina Faso. In addition, there were differences reported in the targets for the key milestones in the full review process, these differences ultimately led to differences in the overall approval times for medicines that were processed via the full review pathway.

Submission of a CPP was also reported as a requirement in the SADC region (18, 19). It was also noted that in the SADC region, “countries with higher workloads had no targets for the scientific assessment or overall approval process” (21). This was not the case for ECOWAS, as all the authorities have targets for the scientific assessment and for the overall approval process. The study in the SADC region also suggested that resources could be optimised by maturing authorities through the use of reliance on more mature authorities (21, 22). This proposition is worth replicating in the ECOWAS region to optimise resources within the sub-region.

In addition, there were differences reported in the targets for the key milestones in the full review process, with Senegal, Ghana and Nigeria reporting a target of 120, 180 and 240 calendar days, respectively. These issues ultimately led to differences in the overall approval times for medicines that were processed via the full review pathway as Ghana, Sierra Leone, Cote d’Ivoire reported different mean approval times of 56, 150, 240 and 247 calendar days, respectively.

### 4.1 Limitation of the study

Although, the NRAs were repeatedly requested to provide the essential information on the questionnaire over the period of the study, the information was not forthcoming partly due to the fact that such data is not captured by the agency which is coupled with the lack of electronic information system. It is however recommended that in a follow up study the manual system is replaced.

### 4.2 Recommendations

The authors’ key recommendations for improving review models and regulatory timelines of countries participating in the ECOWAS-MRH initiative are:

**Publish clients’ service charters—**NRAs should consider publishing their clients’ service charters for regulatory functions that have a timeline component such as registration and marketing authorization, regulatory inspection, licensing establishment and clinical trial’s oversight.

**Recognise the ECOWAS-MRH initiative as a reference authority—**NRAs should adopt regional recommendations of products which have gone through the ECOWAS-MRH joint scientific assessment and expedite granting of marketing authorisation.

**Harmonise Review models and target timelines—**NRAs in the ECOWAS region should consider harmonising their review models and target timelines as this will increase transparency in their regulatory process as well as being of interest to all stakeholders.

**Develop robust information technology systems—**Authorities should invest in robust IT systems to help in the tracking of applications to enable them to be efficient.

**Explore smart ways to communicate to applicants—**Authorities should find innovative ways to effectively communicate with stakeholders to achieve their regulatory mandates on time.

## 5 Conclusion

This comparative study of the review models and regulatory timelines of countries participating in the ECOWAS-MRH initiative has highlighted both the similarities among the authorities and also the gaps that should be addressed in order to improve upon the regulatory systems in these countries.

## Data Availability

The raw data supporting the conclusions of this article will be made available by the authors, without undue reservation.
